# The Secreted Protein Disulfide Isomerase Ag1 Lost by Ancestors of Poorly Regenerating Vertebrates Is Required for *Xenopus laevis* Tail Regeneration

**DOI:** 10.3389/fcell.2021.738940

**Published:** 2021-10-05

**Authors:** Anastasiya S. Ivanova, Maria B. Tereshina, Karina R. Araslanova, Natalia Y. Martynova, Andrey G. Zaraisky

**Affiliations:** ^1^Shemyakin-Ovchinnikov Institute of Bioorganic Chemistry, Russian Academy of Sciences, Moscow, Russia; ^2^Pirogov Russian National Research Medical University, Moscow, Russia

**Keywords:** Agr protein disulfid isomerases, recombinant proteins, tail regeneration, xenopus tadpoles, evolution of regeneration

## Abstract

Warm-blooded vertebrates regenerate lost limbs and their parts in general much worse than fishes and amphibians. We previously hypothesized that this reduction in regenerative capability could be explained in part by the loss of some genes important for the regeneration in ancestors of warm-blooded vertebrates. One of such genes could be *ag1*, which encodes secreted protein disulfide isomerase of the Agr family. *Ag1* is activated during limb and tail regeneration in the frog *Xenopus laevis* tadpoles and is absent in warm-blooded animals. The essential role of another agr family gene, *agr2*, in limb regeneration was demonstrated previously in newts. However, agr*2*, as well as the third member of *agr* family, *agr3*, are present in all vertebrates. Therefore, it is important to verify if the activity of *ag1* lost by warm-blooded vertebrates is also essential for regeneration in amphibians, which could be a further argument in favor of our hypothesis. Here, we show that in the *Xenopus laevis* tadpoles in which the expression of *ag1* or *agr2* was artificially suppressed, regeneration of amputated tail tips was also significantly reduced. Importantly, overexpression of any of these *agrs* or treatment of tadpoles with any of their recombinant proteins resulted in the restoration of tail regeneration in the refractory period when these processes are severely inhibited in normal development. These findings demonstrate the critical roles of *ag1* and *agr2* in regeneration in frogs and present indirect evidence that the loss of *ag1* in evolution could be one of the prerequisites for the reduction of regenerative ability in warm-blooded vertebrates.

## Introduction

Proteins of the anterior gradient (Agr) family belong to the superfamily of protein disulfide isomerases (PDI), all members of which contain the thioredoxin motif and are localized in the endoplasmic reticulum (ER) where they participate in the folding of various proteins (Park et al., 2009; Delom et al., 2020). In contrast to other PDIs, the Agr family, besides operating in the ER, can be secreted in the extracellular space, participating in cell signaling during the embryonic development, in tissue repairing and in cancer (Aberger et al., 1998; Zhu et al., 2017; Moidu et al., 2020).

Two members of *agr* family, *ag1* and *agr2*, were discovered firstly in the frog *Xenopus laevis* (Sive et al., 1989; Aberger et al., 1998; Novoselov et al., 2003). In total, three non-orthologous *agr* genes were identified in vertebrates, *ag1*, *agr2* and *agr3*, which demonstrate closest homology with genes encoding non-secreted PDI of the TLP19 family (Ivanova et al., 2013). Interestingly, whereas *agr2* and *agr3* are present in all vertebrates, *ag1* is specific only for fishes and amphibians (Ivanova et al., 2013).

It was shown that *ag1* in *Xenopus laevis* is involved in the regulation of forebrain development through regulation of the expression of such genes as *foxg1*, *fgf8* and *otx2* (Aberger et al., 1998; Tereshina et al., 2014).

The critical role of *agr2* in limb regeneration was demonstrated in newts (Kumar et al., 2007; Kumar and Brockes, 2012; Grassme et al., 2016). During this, Agr2 operates via binding with its receptor three-finger protein from Ly6 family, Prod1, thus activating the limb blastema cells proliferation (Kumar et al., 2007). It was shown activation of EGF pathway, metalloproteinase MMP9 expression and cell proliferation in the blastema cells during salamander regeneration due to interaction of Prod1, with EGF receptor (Blassberg et al., 2011). Interestingly, Agr2 with a mutation of cysteine in the PDI motif was unable to do so (Grassme et al., 2016). The interaction of Agr2 with the structural and functional homolog of Prod1, Tfp4, was also shown in *Xenopus laevis* (Eroshkin et al., 2017). We demonstrated that Tfp4 is expressed at a low level in the ectoderm of tadpole tail and limb buds, but its expression significantly increased in the regenerative epithelium already on the 1st day after the amputation of these appendages (Tereshina et al., 2019).

In humans, *agr2* is activated in most adenocarcinomas and promotes cell proliferation and cancer progression (Li et al., 2015a; Tsuji et al., 2015; Moidu et al., 2020). A similar role was also demonstrated for *agr3* (Adam et al., 2003; Jian et al., 2020). It was shown that Agr2 stabilized hypoxia-inducible factor-1a HIF1 in breast cancer cells (Li et al., 2015b). Notably, the inhibition of HIF-1α was recently shown to impair regeneration, whereas stabilization of HIF-1α induces regeneration in the refractory period (Ferreira et al., 2018). This finding may indicate a possible mechanism promoting regeneration through stabilization of HIF1α by Agr2. It was also reported that the interaction of Agr2 with the epidermal growth factor receptor and with vascular endothelial growth factor A (VEGF-A) in ER could enhance the activities of these signaling pathways (Dong et al., 2015; Jia et al., 2018).

Despite the role of *agr2* in newt limb regeneration being established (Kumar et al., 2007; Grassme et al., 2016) and elevated expression of *ag1* and *agr2* being shown during the regeneration of the frog (*Xenopus laevis)* tadpoles limbs and tails (Ivanova et al., 2013), it is still unknown whether these two genes also play critical roles in frogs’ regeneration abilities. Additionally, answering this question would contribute to a better understanding of whether the disappearance of *ag1* in the evolutionary younger vertebrate species, in particular in mammals, could be one of the critical reasons for the sharp decline in their ability to regenerate body appendages (Khyeam et al., 2021). Previously, we hypothesized and presented evidence that such a decline, as observed in groups of animals that appeared later in the evolution than amphibians, could be the result of the loss of some genes in their ancestors, which still regulate regeneration in the extant fishes and amphibians (Ivanova et al., 2013, 2015, 2018; Korotkova et al., 2019).

To verify if *ag1* could be one of such genes, we analyzed the effects of *ag1* downregulation and overexpression on the regeneration of *Xenopus laevis* tadpoles’ tails. In addition, we investigated the effects of the downregulation and overexpression of *agr2*, whose role in regeneration in frogs, as far as we know, has not been tested before. As a result, we demonstrate the essential roles of both *ag1* and *agr2* at the cellular and gene expression levels for tail regeneration and blastema cell proliferation. In addition, we found that both overexpression of either of these two genes and treatment of tadpoles with the recombinant protein product of either of them restores regeneration in the refractory period when amputated tail tips cannot regenerate in normal development (Slack et al., 2004). These results confirm the critical role of *ag1* and *agr2* for regeneration in frogs and provide an additional argument in favor of the hypothesis that connects reduction of the regenerative abilities in warm-blooded vertebrates with the loss of some important genes, in particular *ag1*, in their ancestors.

## Materials and Methods

### Manipulations With Tadpoles and Embryos

All experiments with animals were approved by the Animal Committee of the Shemyakin-Ovchinnikov Institute of Bioorganic Chemistry (Moscow, Russia) and the Animals (Scientific Procedures) Act 1986, and the Declaration of Helsinki (Hollands, 1986). Amputation and injections of *Xenopus* tails were performed with MS222 anesthesia.

### Experiments With Morpholino Oligonucleotides

To test the effects of *ag1* and *agr2* downregulation, conventional morpholino oligonucleotides (MOs), vivo-morpholinos (vivo-MOs) and photo-activating morpholinos (photo-MOs)^1^ were used (see Supplementary Figure 1 for MOs structure, specificity and efficiency).

In brief, 4–8 cell embryos were injected in blastomeres, mostly giving rise to the tail bud, with conventional MOs specific to *ag1* and *agr2* mRNAs (4–5 nl of 0.3 mM MO water solution per blastomere) and incubated at 20–22°C until stages 40–42. After tail amputation, tadpoles were incubated at 20–22°C for 1–7 days. Regenerates of 1–7 days were used for regeneration rate analysis, immunochemistry and qRT-PCR. However this approach has one significant weak point. As Agr genes are very important for early development the injections at 4–8 blastomere stages sometimes did not allow to avoid totally Agr MO influence on early development leading to high percent of abnormal and dead tadpoles.

To minimize the possible early effects of conventional MOs, we temporary inactivated them by the complementary photo-MOs, which contained photo-sensitive bonds cleavable with 365 nm light (GeneTools). As a result, *ag1* and *agr2* mRNA translation was not blocked until the embryos were illuminated at the desired stage with 365 nm light, which induces cleavage of photo-MOs and the release of anti-sense MOs. The 4–8 cell embryos were injected (4–5 nl) with 0.3 mM solution of the corresponding anti-sense MO mixed with the sense photo-MO in a dilution of 1:1.3 according to the manufacturer’s recommendations. Importantly, all procedures during and after injections were performed under > 560 nm light (we used a red lamp in a dark room). All embryos were then incubated until stage 40–42 in dark conditions. Before amputation, the tadpoles were exposed for 30 min to 365 nm UV light for activation (releasing) of anti-sense MO. After tail tip amputation, they were incubated at 20–22°C in daylight for 1–7 days. Regenerates of 1–7 days were used for regeneration rate analysis, immunochemistry and qRT-PCR. Photo-MOs are extremely effective, but all procedures with them must be performed under red light, which leads to increased run-off of the MO solution as it was necessary to check the flow from the capillary before each injection. As photo-MOs are used together with conventional MO, experiments with them become quite expensive. Unfortunately, it worth noting that nowadays Gene-Tools no longer produces photo-MO.

In the third approach, we injected fresh tail stumps by the anti-sense vivo-MOs, which can penetrate through plasma membrane due to a unique covalently linked delivery moiety. Thus, local injection of vivo-MO at a certain developmental stage allows one to knock-down the target gene at the desired spatio-temporal parameters. After amputation, the anesthetized tadpoles were transferred from a 0.1 MMR solution (Marc’s Modified Ringer’s solution) with MS322 anesthetic to Petri dishes with a 3% agarose layer. For better spreading of vivo-MO, we injected 0.4 mM solution in a mixture with the fluorescent tracer FLD into the notochord and both fins in the direction of tail growth near the amputated edge immediately after amputation. We repeated injections once per day during 1–4 dpa. After blastema formation, we injected the solution into the fins and notochord and the blastema. On 7–8 dpa, tadpoles with both normally and abnormally regenerated tails were counted. Additionally, at 1–4 dpa, the regenerated tails were collected for immunochemistry, and total RNA extraction was carried out for qRT-PCR.

For statistical analysis, all tails for simplicity were divided into only two categories: (1) regeneration was considered complete, when good regeneration, similar to the regeneration in most control tails, was observed; (2) regeneration was considered defective if the tail did not grow at all or some defects were observed, such as non-regenerating or partially regenerating fins and a curved thin notochord, without surrounding fin blade. In all experiments statistical significance was calculated with *t*-test for independent samples.

### RNA Synthesis and Overexpression Experiments

Synthetic mRNAs were obtained by *in vitro* transcription using mMessage Machine SP6 Kit (Ambion) and *ag1/agr2*-*pCS2* plasmids linearized by Not1. For the over-expression experiments, injections (4–5 nl) of the following concentrations of mRNA were used: *agr2* (300 ng/μl) and *ag1* (300 ng/μl), *ag1* + *agr2* (150 ng/μl + 150 ng/μl). Experiments were performed according to the previously validated method. In brief, we injected 4–8-cell embryos using the solution of the target mRNA and RDA (Rhodamine-labeled dextran-amine) into the blastomeres of the prospective tail buds. The control tadpoles were injected with a water solution of RDA. After tail amputation in the refractory period (stages 46–47), tadpoles were incubated for 1–7 days. Then, regeneration rates were analyzed on morphological (7 dpa), cellular (2–3 dpa) and gene expression levels (0–2 dpa). In total, 400–500 tadpoles were analyzed in three independent experiments for each of control, *ag1* and *agr2* mRNA. To justify this method of ag1 and agr2 overexpression, we demonstrated by qRT-PCR that the injected mRNA presented in tips of tadpoles’ tails during the refractory period in amounts several times higher than the mRNA of the endogenous ag1 and agr2 (Supplementary Figure 2).

### *In situ* Hybridization, Immunostaining and Terminal Deoxynucleotidyl Transferase Mediated dUTP Nick End Labeling Assay

For *in situ* hybridization, we used the protocol described by Harland (1991). To obtain antisense dig-RNA probe for *cyclin D1*, we cloned its cDNA into a pAL2-T vector (Evrogen) and conducted *in vitro* transcription from the PCR-product with dig-NTPs (Roche) and SP6-RNA-polymerase (Thermo Fisher Scientific).

For immunohistochemistry staining, we used the same protocol as previously described in detail (Ivanova et al., 2018). The following antibodies were used: primary rabbit anti-phosphohistone H3 (Millipore, cat. #DAM1545035) (1:100), secondary anti-rabbit CF568 (Sigma, cat. #SAB4600400 and #SAB4600425) (1:500) and anti-rabbit-FITC (Sigma, Cat. # F9887) (1:100). The results were processed by ImageJ software.^2^

The DeadEnd Fluorometric TUNEL System (Promega, Cat. #G3250) was used to reveal the apoptotic cells. The detailed protocol was described previously (Ivanova et al., 2015).

During these experiments, we determined the border between the old and regenerating part of the tail taking advantage of the fact that even after 5–7 dpa, the regenerating part has more transparent notochord and less structured muscles.

### qRT-PCR

qRT-PCR was performed and evaluated as described previously in Ivanova et al. (2013). Briefly, for total RNA extraction from the regenerating tail tips (1–4 dpa) and isolation, we used, respectively, an RNA extract reagent (Evrogen) and RNA isolation KIT (Evrogen). About 20–30 tails were used for each sample for total RNA extraction. For both MO injections and controls, we took tail’s’ tip tissues, cutting off a piece of the stump proximally to the amputation level extending at a distance of 1/4–1/5 of the tail width from the amputation level. To equalize the amount of tissue, we usually used 5–10 more tails in experimental samples than in the control ones. The RNA quality and concentration were measured by NanoPhotometer N60 (Implen). The reverse transcription (RT) of purified RNA samples was carried out using the M-MLV reverse transcriptase kit (Evrogen) according to the manufacturer’s guidelines. The qPCR with marker primers (see Supplementary Material) and the qPCR-mix HS SYBR (Evrogen) were conducted on the DTprime 4 qPCR amplifiers (DNA-Technology) with a standard 40-cycle hot start program. The obtained PCR data were calculated using the ΔΔCt method. The geometric mean of expression of ODC and EF-1alpha (housekeeping genes) was used for the normalization of gene expression levels. The normalized PCR signal of the 0 dpa sample was taken as an arbitrary unit (a.u.) in each series. The data for each gene expression were calculated in 3–7 independent experiments.

### Treatment of Tadpoles With Recombinant Ag1 and Agr2 Proteins

After amputation of the tips of the tails, the tadpoles were incubated in 50 ml Petri dishes with 0.1 MMR, to which recombinant Ag1 and Agr2 were added once to a final concentration of 3 μg/ml (see Supplementary Materials and Supplementary Figure 3 for the procedure of the purification of the recombinant proteins and testing their integrity at successive days after addition to the medium with tadpoles). The same final concentration of BSA was used in the control experiments.

## Results

### Downregulation of *Ag1 or Agr2*, or Both, Suppresses Tail Regeneration

Previously, we demonstrated that both in the amputated tails and hind limb buds of *Xenopus laevis* tadpoles, the expression levels of *ag1* and *agr2* had strongly increased on the first day postamputation (1 dpa), reaching a maximum on the 2 dpa, and gradually decreased afterward (Ivanova et al., 2013). Using the regeneration of the amputated tails as a model, we then decided to verify if such activation of the expression of these two genes is necessary for successful tail regeneration.

To this end, we arranged a series of experiments in which we investigated the effects on tail regeneration of the downregulation of *ag1* and *agr2*, alone or together, provoked by the antisense morpholino oligonucleotides (MO) to their mRNA. To achieve the greatest reliability, we used three different types of MOs: conventional MO, photo-MO and vivo-MO.

We injected the conventional MO into the blastomeres of 4–8 cell-stage embryos, which in most cases gives rise to tadpoles’ tails. To minimize the likelihood of possible early effects of the conventional MO, which could have long-term consequences, thus affecting tail regeneration, we downregulated *ag1* and *agr2* by an photo-inducible morpholinos using mixture of anti-sense conventional MO with sense photo-MOs (further named photo-MO). Although photo MOs were injected into the early embryos in the same way as conventional MOs, they were inactive almost until the tail amputation, when we activated them by 365 nm blue light (see “Materials and Methods” section for details). Finally, to avoid any manipulation of the embryos until the tadpole stage, we used *ag1* and *agr2* vivo-MOs, which can penetrate through plasma membrane due to a unique covalently linked delivery moiety. In these experiments, we injected vivo-MOs directly into the tail tips immediately after amputation.

**FIGURE 1:**
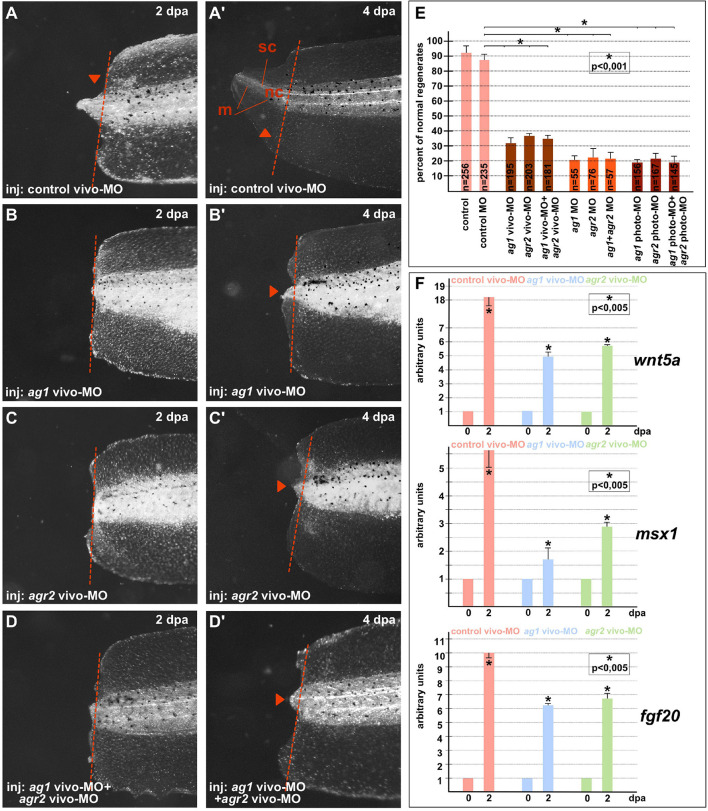
Downregulation of ag1and/or agr2 genes leads to regeneration blockage. **(A,A’)** Imaging of *Xenopus laevis* tadpoles developed from embryos injected with control morpholino oligonucleotides (control MO) and regenerating tail tip at two developmental timepoints corresponding to 2 and 4 days post amputation (dpa). Lateral view, dorsal to the top. Dashed red line indicates amputation level. Sc, spinal cord; nt, neural tube; m, muscles. Tail tip regeneration is dramatically reduced if *ag1* and/or *agr2* genes are downregulated by injection of embryos with *ag1* vivo-MO **(B,B’)**, *agr2* vivo-MO **(C,C’)**, or both **(D,D’)**. **(E)** Quantification of normal regenerates percentage among controls and *ag1/agr2* morphants. N—number of tails analyzed. Error bars indicate SD. Statistical significance was determined with *t*-test for independent samples; the results are statistically significant, *p* < 0.001 (asterisk). **(F)** qRT-PCR analysis of expression levels changes of regeneration markers *wnt5a, msx1* and *fgf20* during the regeneration process (at 0 and 2 dpa) in amputated tails of tadpoles injected with *control*, *ag1* and/or *agr2 vivo-MO*. The value of normalized PCR signal in the 0 dpa sample, harvested immediately after amputation, was taken as an arbitrary unit in each series. Dpa—days post amputation. Error bars indicate SD, *t*-test, *p* < 0.05 (asterisk).

On 4–5 dpa, we scored the regeneration effectiveness in each experimental group, comparing it with that in the groups of the control sibling embryos injected with the control MOs (Figures 1A–E). As a result, we established that whereas in all control (control and control MO) groups there were about 90–95% of normally regenerating tails, the percentage of such tails was dramatically lower in the groups of tadpoles in which *ag1* and *agr2* were downregulated alone or together. In these groups, the percentage of normally regenerating tails varied from 20 to 38% depending on the type of MOs (Figure 1E). We also confirmed these effects of *ag1* and *agr2* downregulation by revealing at 2 dpa in the amputated tail tip tissues a strong decrease in the expression of three essential regulators of tail regeneration, *fgf20*, *msx1b* and *wnt5a* (Beck et al., 2003; Lin and Slack, 2008; Figure 1F).

All these results confirm that the activity of *ag1* and *agr2* during the first days after amputation is essential for tail regeneration.

### *Ag1 and Agr2* Downregulation Suppresses Cell Proliferation but Does Not Affect Apoptosis in the Regenerating Tail Tissues

As was discovered, downregulation of *ag1* and *agr2* resulted in a significant shortening of tail regenerates or the absence of growth or elongation. To verify if these effects were the result of cell proliferation inhibition, apoptosis activation, or both, we compared cell proliferation and apoptosis in the regenerating tail tips of the control tadpoles and those in which *ag1* and *agr2* were downregulated by photo- and/or vivo-MOs.

**FIGURE 2:**
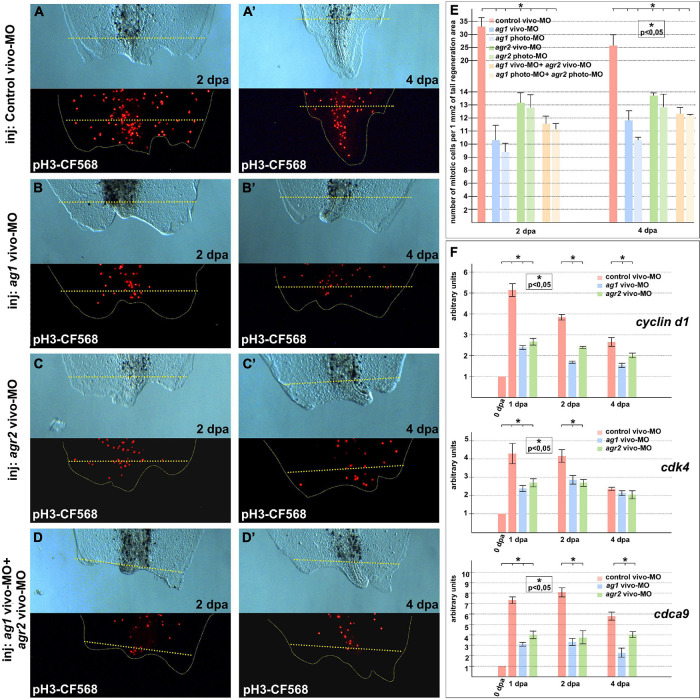
Cell proliferation is inhibited in regenerating tails under *ag1* and/or *agr2* downregulation conditions. **(A,A’)** The transmitted light and fluorescent images of regenerating tails of tadpoles injected with solution of control vivo-MO after immunostaining with primary rabbit anti-pH3 and secondary anti-rabbit antibodies conjugated with red fluorescent protein CF568 demonstrate mitotic activity in the regenerating area at 2 and 4 dpa, respectively (see **E** for statistics). Transmitted light and immunostained fluorescent images of tadpoles injected with *ag1* vivo-MO **(B,B’)**, *agr2* vivo-MO **(C,C’)**, or a mixture of them **(D,D’),** show strong inhibition of mitotic activity at 2 and 4 dpa. Dashed yellow line indicates amputation level. **(E)** Quantification of number of mitotic cells per 1 mm2 of tail regenerating area. Data of five independent experiments (10 tadpoles of each injection type were used in 1 experiment) were used for statistical analysis; statistical significance was determined by *t*-test for independent samples, *p* < 0.05 (asterisk). Error bars indicate SD. **(F)** qRT-PCR analysis of expression levels changes of cell cycle markers *cyclin d1, cdk4*, and *cdca9* during the regeneration process (at 0, 1, 2, and 4 dpa) in amputated tails of tadpoles injected with *control*, *ag1* and/or *agr2* vivo-MO. The value of normalized PCR signal in the 0 dpa sample, harvested immediately after amputation, was taken as an arbitrary unit in each series. Dpa—days post amputation. Error bars indicate SD, *t*-test, *p* < 0.05 (asterisk).

When we had analyzed the mitotic activity using monoclonal antibodies to the specific marker of the S-phase, phosphor-histone 3B, a decrease in the number of mitotic cells in the 1–2 dpa regenerating tails with downregulated *ag1*, *agr2*, or both, as compared to the control tails, was detected. Notably, the effect was more pronounced in tails injected with *ag1* MOs (Figures 2A–E). In some of the tails with downregulated *ag1* or *agr2*, we observed that the number of dividing cells in the tail area near the amputation plane was much less than in the control tails (compare Figures 2A,A’ with Figures 2B–D’). During early period of regeneration (1–4 dpa), an intensive epithelial cell proliferation covering the injury followed by dedifferentiation and proliferation of blastema cells took place (Tseng and Levin, 2008). In support of the critical roles of *ag1* and *agr2* for cell proliferation, we also revealed by qRT-PCR a statistically significant decrease in the expression levels of several cell cycle regulatory genes, c*yclin D1*, *cdk4* (*cyclin-dependent kinase 4*) and *cdca9* (*cell division cycle-associated 9*) (Sampath et al., 2004; Musgrove et al., 2011), in the regenerating tail tissues of tadpoles at 1–4 dpa with downregulated *ag1*, *agr2*, or both, as compared to the control vivo-MO tails (Figure 2F).

**FIGURE 3:**
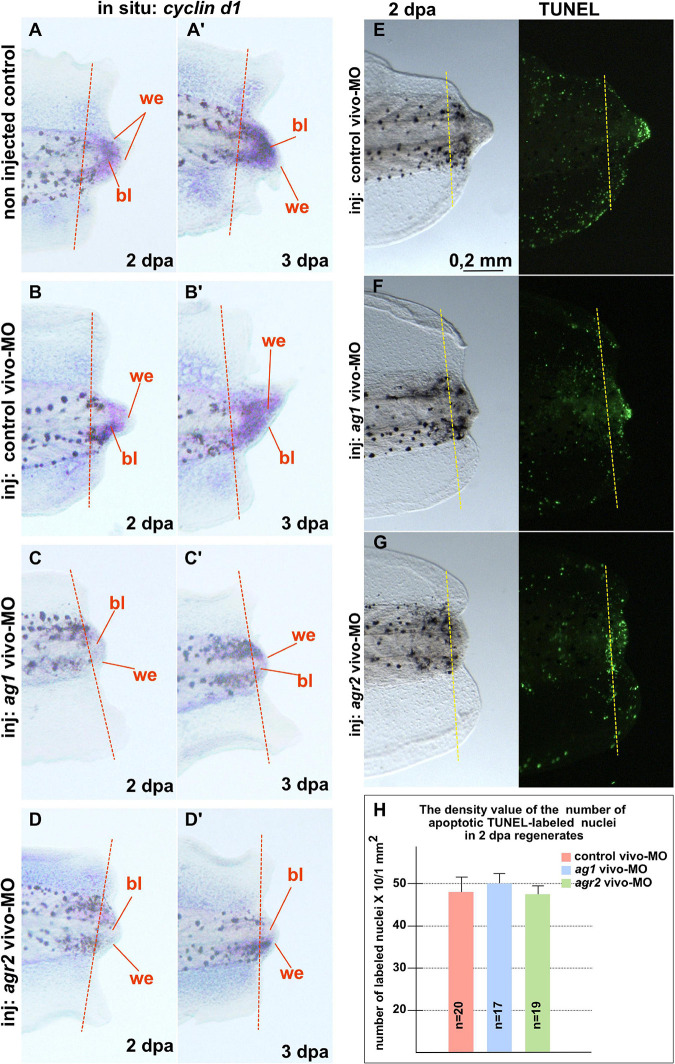
Downregulation of *ag1* and *agr2* during regeneration is accompanied by cell cycle regulator *cyclin d1* expression blockage but does not affect apoptosis activity in the regenerating tail. **(A,A’)**. Results of *in situ* hybridization of non-injected control regenerating tails as well as tails injected with control vivo-MO **(B,B’)** demonstrate active expression of *cyclin D1* at 2 and 3 dpa predominantly in blastema cells. Knock-down of *ag1* **(C,C’)** as well as *agr2* **(D,D’)** by specific vivo-MOs result in a high reduction of *cyclin d1* expression at 2 and 3 dpa. Bl—blastema, we—wound epithelium. Dashed red line indicates amputation level. Lateral view, distal to the right. **(E–G)** TUNEL analysis of apoptotic cells pattern in 2 dpa regenerating tails injected with control vivo-MO **(E)**, *ag1* vivo-MO **(F)** or *agr2* vivo-MO **(G)**. **(H)** Statistical analysis of number of TUNEL-labeled nuclei per 1 mm^2^ of regenerating region, distal to the amputation level (yellow dashed line). N—number of tails analyzed.

In addition, we examined changes in the spatial pattern of *cyclin D1* expression by *in situ* hybridization on regenerates with normal and downregulated expression of *ag1/agr2*. During normal regeneration, *cyclin D1* mRNA was clearly detected in the formation of blastemas at 2 and 3 dpa (Figures 3A–B’). This spatiotemporal pattern of c*yclin D1* expression correlates well with the data of intensive cell proliferation of dedifferentiated cells in the forming blastema. However, in the tails injected with *ag1*/*agr2* MO, c*yclin D1* expression was significantly reduced (Figures 3C–D’).

Thus, we concluded that the activities of *ag1* and *agr2* are necessary for active cell proliferation in the regenerating tails.

Then, to test if the increased cell death could also give rise to the suppression of tail regeneration in tadpoles with downregulated *ag1* and *agr2*, we investigated patterns of the apoptotic cells in regenerating tadpole tails injected with *ag1*, *agr2*, or control vivo-MO, using the TUNEL assay. However, we could not find statistically significant differences in the mean density of apoptotic cells after injections of ag1/agr2 vivo-MO between normally regenerating tails and tails with suppressed regeneration, neither in the regenerating tips themselves nor the regions proximal to the level of amputation (Figures 3E–H and Supplementary Figure 4). These results indicate that the suppression of tail regeneration caused by downregulation of *ag1* and *agr2* was not the result of changes in the normal intensity of apoptosis.

### Overexpression of *Ag1* and *Agr2* Can Unlock the Blockage of Regeneration in the Refractory Period

Despite *Xenopus laevis* tadpoles, in general, being able to regenerate amputated tails, there is a special refractory period between stages 45 and 47, during which the regeneration ability is temporarily blocked (Slack et al., 2004). While initial causes of this blockage are not completely known, it was shown that critical processes for the earliest steps of regeneration processes such as reactive oxygen species production, activation of the HIF-1α pathway and recruitment of innate immune cells to the injury site (such as macrophages) are downregulated in this period (Fukazawa et al., 2009; Love et al., 2013; Ferreira et al., 2018). Activation of these processes by various experimental cues was shown to be sufficient for the activation of tail regeneration in the refractory period. Moreover, downregulation in this period of several other late regulators of tail regeneration was reported, and the activation of regeneration in case of their overexpression was also demonstrated (Tseng et al., 2010; Kakebeen and Wills, 2019). Notably, we previously demonstrated the same for two proteins, whose genes were lost during evolution in poorly regenerating higher vertebrates: for small GTPase Ras-dva1 and the transmembrane modulator of FGF and purinergic signaling, c-Answer (Ivanova et al., 2018; Korotkova et al., 2019).

**FIGURE 4:**
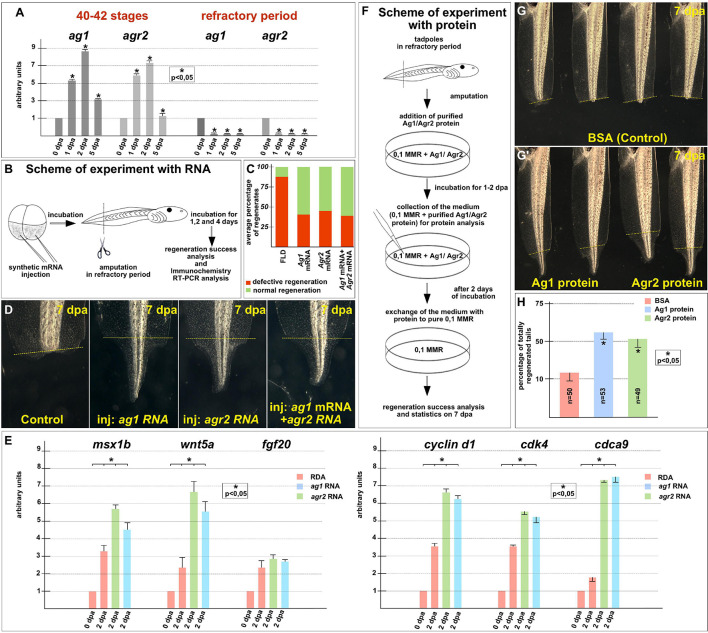
Regeneration blockage during tadpoles’ refractory period can be unlocked by *ag1/agr2* over-expression or incubation in solution with purified Ag1/Agr2 proteins. **(A)** The qRT-PCR results show the difference in expression dynamics of *ag1* and *agr2* at 0–5 dpa in tadpole regenerates upon amputation at stage 40–42 or in the refractory period. The value of normalized PCR signal in the 0 dpa sample, harvested immediately after amputation, was taken as an arbitrary unit in each series. Dpa—days post amputation. Error bars indicate SD, *t*-test, *p* < 0.05 (asterisk). **(B)** Scheme of the experiment with *ag1/agr2* over-expression and types of analysis of the regeneration process in the refractory period. **(C)** Statistical analysis of regeneration success of tadpoles, developed from embryos injected with a solution of FLD, either *ag1* or *agr2* mRNA (or both), and amputated in the refractory period. The picture shows the average values. **(D)** The transmitted light images of tails of corresponding tadpoles on 7 day post amputation in the refractory period demonstrate total regeneration of tails in tadpoles over-expressing either *ag1* or *agr2* mRNA (or both). **(E)** qRT-PCR analysis of expression levels changes of regeneration markers *msx1b*, *wnt5a*, and *fgf20* and cell cycle markers *cyclin d1*, *cdk4* and *cdca9* during the regeneration process (at 0 and 2 dpa) in amputated tails of tadpoles injected with RDA, *ag1* or *agr2* mRNA solution. The value of normalized PCR signal in the 0 dpa sample, harvested immediately after amputation, was taken as an arbitrary unit in each series. Dpa—days post amputation. Error bars indicate SD, *t*-test, *p* < 0.05 (asterisk). **(F)** Scheme of the experiment with tadpoles amputated in refractory period and incubated in solution with BSA, Ag1 or Agr2 purified proteins (see Supplementary Material for the procedure of the recombinant proteins preparation and Supplementary Figure 3B for testing the integrity of the proteins in the medium with tadpoles). **(G,G’)** The transmitted light images of tadpoles tails on day 7 post amputation in the refractory period after incubation in BSA solution **(G)** or in solution with Ag1 or Agr2 proteins **(G’)** demonstrate total regeneration only in the latter variants. **(H)** Statistics of normally regenerated tails percentage among tadpoles amputated in refractory period and incubated with BSA or purified Ag1 or Agr2 proteins. N—total number of tadpoles used in three independent experiments.

To arrange similar testing for *ag1* and *agr2*, we first compared their normal expression dynamics after tail amputation was performed before the refractory period, at stages 40–42, with the expression dynamics when the amputation was performed directly during this period, at stages 45–47. Consistent with our previous results (Ivanova et al., 2013), we found that before the refractory period, the expression of *ag1* and *agr2* strictly increased by six to nine times on the first and second days after amputation (Figure 4A). At the same time, in the tails amputated during the refractory period, the expression of both these genes remained at a low level during at least 5 dpa (Figure 4A).

Then, we tested whether tail regeneration in the refractory period could be initiated by overexpression of the *ag1* and/or *agr2*. Indeed, when we overexpressed any of these genes in tadpoles by a previously validated method of injecting synthetic mRNA in the tailbud precursor blastomeres of embryos in stage 4–8 blastomeres (Ivanova et al., 2018), we obtained results clearly confirming the ability of the overexpressed *ag1* and *agr2* to rescue tail regeneration in refractory period (Figures 4B–E). Thus, if in the control groups 85–88% of the amputated tails did not regenerate at all or were with various defects, and only 12–15% normally regenerated, in the groups of tadpoles injected with *ag1* mRNA, only 35–45% of tails regenerate with defects or not regenerate and 55–65% regenerated normally. Similar results were obtained for the amputated tails overexpressing *agr2* mRNA or a mixture of *ag1* and *agr2* mRNAs: the corresponding values were 32–54% and 46–58% in the first case and 35–40% and 60–65% in the second (Figures 4C,D).

Importantly, after analyzing tails overexpressing *ag1* and *agr2*, which were amputated in the refractory period, the expression of two regulators of tail regeneration, *msx1b* and *wnt5a*, was increased compared to the control amputated tails (Figure 4E). These results indicate that overexpression of *ag1* and *agr2* is sufficient to induce regeneration in the refractory period leading to the activation of at least some key signaling pathways that normally regulate regeneration.

The analysis of the proliferative status also showed similar results to the normal regeneration increase in the expression of cell cycle markers, *cyclin D1, cdk4* and *cdca9* and the mitotic index in the amputated refractory tails of tadpoles overexpressing *ag1* and *agr2* compared with the control ones (Figure 4E and Supplementary Figure 5).

The data obtained suggest that Agr proteins restore regeneration ability during the refractory period by activating, directly or indirectly, mitotic activity and signaling pathways essential for regeneration.

### Ag1 and Agr2 Recombinant Proteins Can Reactivate Tail Regeneration in the Refractory Period Indicating Their Direct Influence Upon Stump Cells

Since in the experiments described above we activated regeneration in refractory tails by injecting *ag1* and *agr2* mRNAs into early embryos, it remained unclear whether such activation was actually caused by the direct influence of these proteins on the stump cells or whether it was a result of some of their actions in earlier stages. In addition, in these experiments, it was impossible to distinguish which of the two possible modes of action of Ag1 and Agr2 was decisive for the activation of regeneration: from the outside or from the inside of cells. As is known, Agrs can operate either in the endoplasmic reticulum or the Golgi apparatus, where they perform the function of chaperones, changing the conformation of proteins, including, possibly, some signaling factors essential for regeneration, or, they can be secreted from the cell, executing functions of such signaling factors themselves (Maurel et al., 2019; Delom et al., 2020).

To determine whether Ag1 and Agr2 could act directly from the outside of cells of the refractory stumps, we arranged experiments in which we treated the stumps with the recombinant Ag1 and Agr2 proteins. In these experiments, we added purified recombinant Ag1 and Agr2, or BSA as a control, in the final concentration of 3 μg per ml (see Supplementary Materials for details of how they were obtained and purified) to the refractory tadpoles (stage 46) kept in 0.1 × MMR, immediately after tail tip amputation. At 2 dpa, the proteins containing the mediums were changed for 0.1 × MMR, and tadpoles were incubated at room temperature until 5 dpa when the regeneration efficiency was scored as compared to control (Figure 4F).

As a result, we detected an evident increase in the tail regeneration frequency in the groups of tadpoles treated by the recombinant Agr proteins. Thus, if there were only 10–15% of regenerating tails in the control group, in the groups of tadpoles treated by Ag1 or Agr2, we revealed 55 and 50% of complete regenerates, respectively (Figures 4G,H). These results indicate that Ag1and Agr2 can activate tail regeneration in the refractory period by influencing the stump cells from the outside.

## Discussion

In this work, we have presented the following line of evidence confirming that both *ag1* and *agr2* are important for tail regeneration in *Xenopus laevis*.

First, suppression of any of these genes by any of the three types of antisense morpholinos, namely, ordinary MO, vivo-MO and photo-MO, resulted in the suppression of tail regeneration, accompanied by the suppression of blastema cell proliferation and downregulation of the regeneration marker genes. Importantly, the fact that distinct suppression of regeneration was observed when the MO activity was switched on just before tail amputation (vivo-MO and photo-MO) confirms that the effect was indeed the result of *ag1* or *agr2* downregulation.

Second, the overexpression of *ag1* or *agr2* in tadpoles, which was achieved by microinjection of mRNA encoding these proteins into embryos, resulted in the re-activation of tail regeneration in the refractory period. Concomitantly, enhanced regeneration genetic markers and cell proliferation was observed in the tips of the amputated tails of these tadpoles.

Excitingly, it also appeared to be possible to induce regeneration in the refractory period by treating tadpoles after amputation of their tail tips with any of the recombinant *ag1* and *agr2* proteins. The latter result is critically important because it confirms the specificity of the artificial enhancement of *ag1* and *agr2* levels for the re-activation of regeneration in the refractory period. Previously, only the entry into the S phase of newt blastema cells growing in culture after the addition to this culture of *agr2* recombinant protein was shown (Grassme et al., 2016). The results of our experiments demonstrate that even complete tail regeneration can be triggered by the treatment of tadpoles with Agr proteins.

It was established earlier that during limb regeneration in newts, *agr2* is secreted at first by Schwann cells of the limb nerve sheath (Kumar et al., 2007). In turn, *agr2* secreted by these cells induces its own expression in the secretory cells of the regenerative epithelia covering the wound (Kumar and Brockes, 2012). The *agr2* expression in these secretory cells is absolutely critical because it triggers all processes of regeneration, including blastema growth (Kumar et al., 2007; Kumar and Brockes, 2012). In addition, the authors of cited papers revealed that during this Agr2 may operate through its receptor Prod1.

According to the recently published atlas of single-cell transcriptomics, both *ag1* and *agr2* are also expressed during the regeneration of the *Xenopus laevis* tadpole tails in the epithelial secretory cells (Aztekin et al., 2019). However, their expression is not detected in the population of cells of the wound epithelia, which was shown in the same work to play a primary role in governing tail regeneration, i.e., in regeneration organizer cells (ROC). The latter cells specifically produce many signaling factors, in particular, Bmp2, Bmp4, Fgf4, Fgf7, Fgf9, Fgf10, Wnt3a, Wnt5a, and Wnt7b, whose activities are necessary for regeneration (Aztekin et al., 2019). Therefore, one may predict that in case of stimulation regeneration via modifying the activity of the aforementioned factors or their receptors, Ag1 and Agr2 diffusing from secretory epithelial cells should either interact with these factors directly in the intercellular space or change their synthesis by influencing ROC or other cells also from the intercellular space. Also, Agrs may operate via the *Xenopus* homolog of Prod1, their own receptor Tfp4, which is abundantly expressed in the regenerative epithelia (Tereshina et al., 2019). In turn, this may indicate that the recombinant Ag1 and Agr2 in our experiments could stimulate the regeneration in a similar manner, i.e., by modifying the activities of the aforementioned signaling factors, their receptors or operating through Tfp4 in the intercellular space. To confirm these predictions, it would be important in the future to test if recombinant *ag1* and *agr2* are able to rescue tail regeneration in the context of endogenous *ag1* and *agr2* downregulated by anti-sense morpholinos.

As we have established, the downregulation of even one of the two tested Agr genes appeared to be sufficient to suppress tadpole tail regeneration. This result indirectly confirms that the loss of *ag1* alone in the ancestors of warm-blooded animals could be one of the reasons that led to the decline in their regenerative potencies. Earlier, we demonstrated essential roles of two other proteins for the regeneration of body appendages in fishes and amphibians, whose genes were lost in warm-blooded animals: small GTPase Ras-dva and transmembrane modulator of FGF and purinergic signaling c-Answer (Ivanova et al., 2018; Korotkova et al., 2019). Thus, the present work reveals the critical role of *ag1* for frog tadpole tail regeneration and provides one more argument in favor of our hypothesis that connects the reduction of regenerative abilities in the warm-blooded animals with the loss of some important genes in their ancestors.

## Data Availability Statement

The original research results and data are presented in the article and in Supplementary Material. Additional requests can be sent to the author.

## Ethics Statement

The animal study was reviewed and approved by Animal Committee of the Shemyakin-Ovchinnikov Institute of Bioorganic Chemistry (Moscow, Russia). Written informed consent was obtained from the owners for the participation of their animals in this study.

## Author Contributions

AI, KA, and MT performed experiments with MO injections, *in situ* hybridization, immunohistochemistry and qRT-PCR. NM obtained recombinant proteins and analyzed protein quality. AI, MT, and AZ oversaw all aspects of the manuscript, conceived and designed the project, and wrote the manuscript. AZ generated the initial idea of the project. All authors contributed to the manuscript and have approved the submitted version.

## Conflict of Interest

The authors declare that the research was conducted in the absence of any commercial or financial relationships that could be construed as a potential conflict of interest.

## Publisher’s Note

All claims expressed in this article are solely those of the authors and do not necessarily represent those of their affiliated organizations, or those of the publisher, the editors and the reviewers. Any product that may be evaluated in this article, or claim that may be made by its manufacturer, is not guaranteed or endorsed by the publisher.

## Footnotes

1
Gene-tools.com

2
rsb.info.nih.gov/ij

## References

[B1] Aberger F. Gilbert W. Horst G. Klaus R. (1998). Anterior specification of embryonic ectoderm: the role of the *Xenopus* cement gland-specific gene XAG-2. *Mech. Dev*. 72 115–130. 10.1016/S0925-4773(98)00021-5 9533957

[B2] Adam P. Boyd R. Tyson K. L. Graham C. F. Stamps A. Hudson L. (2003). Comprehensive proteomic analysis of breast cancer cell membranes reveals unique proteins with potential roles in clinical cancer. *J. Biol. Chem*. 278 6482–6489. 10.1074/jbc.M210184200 12477722

[B3] Aztekin C. Hiscock T. W. Marioni J. C. Gurdon J. B. Simons B. D. Jullien J. (2019). Identification of a regeneration-organizing cell in the *Xenopus* tail. *Science* 364 653–658. 10.1126/science.aav9996 31097661 PMC6986927

[B4] Beck C. W. Christen B. Slack J. M. W. (2003). Molecular pathways needed for regeneration of spinal cord and muscle in a vertebrate. *Dev. Cell* 5 429–439. 10.1016/S1534-5807(03)00233-8 12967562

[B5] Blassberg R. A. Garza-Garcia A. Janmohamed A. Gates P. B. Brockes J. P. (2011). Functional convergence of signalling by GPI-anchored and anchorless forms of a salamander protein implicated in limb regeneration. *J. Cell Sci.* 124 47–56. 10.1242/jcs.076331 21118959 PMC3001407

[B6] Delom F. Mohtar M. A. Hupp T. Fessart D. (2020). The anterior gradient-2 interactome. *Am. J. Physiol. Cell Physiol.* 318 C40–C47. 10.1152/ajpcell.00532.2018 31644305

[B7] Dong A. Wodziak D. Lowe A. W. (2015). Epidermal growth factor receptor (EGFR) signaling requires a specific endoplasmic reticulum thioredoxin for the post-translational control of receptor presentation to the cell surface. *J. Biol. Chem.* 290 8016–8027. 10.1074/jbc.M114.623207 25666625 PMC4375459

[B8] Eroshkin F. M. Martynova N. Y. Bayramov A. V. Ermakova G. V. Ivanova A. S. Korotkova D. D. (2017). Interaction of secreted factor Agr2 with its potential receptors from the family of three-finger proteins. *Russ. J. Bioorg. Chem.* 43 344–346. 10.1134/S1068162017030049

[B9] Ferreira F. Raghunathan V. K. Luxardi G. Zhu K. Zhao M. (2018). Early redox activities modulate *Xenopus* tail regeneration. *Nat. Commun.* 9:4296. 10.1038/s41467-018-06614-2 30327466 PMC6191437

[B10] Fukazawa T. Naora Y. Kunieda T. Kubo T. (2009). Suppression of the immune response potentiates tadpole tail regeneration during the refractory period. *Development* 136 2323–2327. 10.1242/dev.033985 19515697

[B11] Grassme K. S. Garza-Garcia A. Delgado J. P. Godwin J. W. Kumar A. Gates P. B. (2016). Mechanism of action of secreted newt anterior gradient protein. *PLoS One* 11:e0154176. 10.1371/journal.pone.0154176 27100463 PMC4839744

[B12] Harland R. M. (1991). In situ hybridization: an improved whole-mount method for *Xenopus* embryos. *Methods Cell Biol.* 36 685–695. 10.1016/s0091-679x(08)60307-6 1811161

[B13] Hollands C. (1986). The animals (scientific procedures) act 1986. *Lancet* 328 32–33. 10.1016/S0140-6736(86)92571-7 2873327

[B14] Ivanova A. S. Korotkova D. D. Ermakova G. V. Martynova N. Y. Zaraisky A. G. Tereshina M. B. (2018). Ras-Dva small GTPases lost during evolution of amniotes regulate regeneration in anamniotes. *Sci. Rep.* 8:13035. 10.1038/s41598-018-30811-0 30158598 PMC6115384

[B15] Ivanova A. S. Shandarin I. N. Ermakova G. V. Minin A. A. Tereshina M. B. Zaraisky A. G. (2015). The secreted factor Ag1 missing in higher vertebrates regulates fins regeneration in Danio Rerio. *Sci. Rep.* 5:8123. 10.1038/srep08123 25630240 PMC4309956

[B16] Ivanova A. S. Tereshina M. B. Ermakova G. V. Belousov V. V. Zaraisky A. G. (2013). Agr genes, missing in amniotes, are involved in the body appendages regeneration in frog tadpoles. *Sci. Rep.* 3:1279. 10.1038/srep01279 PMC3573343 23412115

[B17] Jia M. Guo Y. Zhu D. Zhang N. Li L. Jiang J. (2018). Pro-metastatic activity of AGR2 interrupts angiogenesis target bevacizumab efficiency via direct interaction with VEGFA and activation of NF-K B pathway. *Biochim. Biophys. Acta* 1864 1622–1633. 10.1016/j.bbadis.2018.01.021 29410027

[B18] Jian L. Xie J. Guo S. Yu H. Chen R. Tao K. (2020). AGR3 promotes estrogen receptor-positive breast cancer cell proliferation in an estrogen-dependent manner. *Oncol. Lett*. 20 1441–1451. 10.3892/ol.2020.11683 32724387 PMC7377037

[B19] Kakebeen A. D. Wills A. E. (2019). More than just a bandage: closing the gap between injury and appendage regeneration. *Front. Physiol.* 10:81. 10.3389/fphys.2019.00081 30800076 PMC6376490

[B20] Khyeam S. Lee S. Huang G. N. (2021). Genetic, epigenetic, and post-transcriptional basis of divergent tissue regenerative capacities among vertebrates. *Adv. Genet.* 2 1–14. 10.1002/ggn2.10042 34423307 PMC8372189

[B21] Korotkova D. D. Lyubetsky V. A. Ivanova A. S. Rubanov L. I. Seliverstov A. V. Zverkov O. A. (2019). Bioinformatics screening of genes specific for well-regenerating vertebrates reveals c-answer, a regulator of brain development and regeneration. *Cell Rep.* 29 1027–1040.e6. 10.1016/j.celrep.2019.09.038 31644900 PMC6871517

[B22] Kumar A. Brockes J. P. (2012). Nerve dependence in tissue, organ, and appendage regeneration. *Trends Neurosci*. 35 691–699. 10.1016/j.tins.2012.08.003 22989534

[B23] Kumar A. Godwin J. W. Gates P. B. Garza-Garcia A. Brockes J. P. (2007). Molecular basis for the nerve dependence of limb regeneration in an adult vertebrate. *Science* 318 772–777. 10.1126/science.1147710 17975060 PMC2696928

[B24] Li Z. Wu Z. Chen H. Zhu Q. Gao G. Hu L. (2015a). Induction of anterior gradient 2 (AGR2) plays a key role in insulin-like growth factor-1 (IGF-1)-induced breast cancer cell proliferation and migration. *Med. Oncol.* 32 1–12. 10.1007/s12032-015-0577-z 25956506 PMC4451465

[B25] Li Z. Zhu Q. Hu L. Chen H. Wu Z. Li D. (2015b). Anterior gradient 2 is a binding stabilizer of hypoxia inducible factor-1α that enhances CoCl2-induced doxorubicin resistance in breast cancer cells. *Cancer Sci.* 106 1041–1049. 10.1111/cas.12714 26079208 PMC4556394

[B26] Lin G. Slack J. M. W. (2008). Requirement for Wnt and FGF signaling in *Xenopus* tadpole tail regeneration. *Dev. Biol.* 316 323–335. 10.1016/j.ydbio.2008.01.032 18329638

[B27] Love N. R. Chen Y. Ishibashi S. Kritsiligkou P. Lea R. Koh Y. (2013). Amputation-induced reactive oxygen species are required for successful *Xenopus* tadpole tail regeneration. *Nat. Cell Biol.* 15 222–228. 10.1038/ncb2659 23314862 PMC3728553

[B28] Maurel M. Obacz J. Avril T. Ding Y.-P. Papadodima O. Treton X. (2019). Control of anterior GR adient 2 (AGR 2) dimerization links endoplasmic reticulum proteostasis to inflammation. *EMBO Mol. Med.* 11 1–19. 10.15252/emmm.201810120 31040128 PMC6554669

[B29] Moidu N. A. Rahman N. S. A. Syafruddin S. E. Low T. Y. Mohtar M. A. (2020). Secretion of pro-oncogenic AGR2 protein in cancer. *Heliyon* 6:e05000. 10.1016/j.heliyon.2020.e05000 33005802 PMC7519367

[B30] Musgrove E. A. Caldon C. E. Barraclough J. Stone A. Sutherland R. L. (2011). Cyclin D as a therapeutic target in cancer. *Nat. Rev. Cancer* 11 558–572. 10.1038/nrc3090 21734724

[B31] Novoselov V. V. Alexandrova E. M. Ermakova G. V. Zaraisky A. G. (2003). Expression zones of three novel genes abut the developing anterior neural plate of *Xenopus* embryo. *Gene Expr. Patterns* 3 225–230. 10.1016/S1567-133X(02)00077-7 12711553

[B32] Park S. W. Zhen G. Verhaeghe C. Nakagami Y. Nguyenvu L. T. Barczak A. J. (2009). The protein disulfide isomerase AGR2 is essential for production of intestinal mucus. *Proc. Natl. Acad. Sci. U.S.A*. 106 6950–6955. 10.1073/pnas.0808722106 19359471 PMC2678445

[B33] Sampath S. C. Ohi R. Leismann O. Salic A. Pozniakovski A. Funabiki H. (2004). The chromosomal passenger complex is required for chromatin-induced microtubule stabilization and spindle assembly. *Cell* 118 187–202. 10.1016/j.cell.2004.06.026 15260989

[B34] Sive H. L. Hattori K. Weintraub H. (1989). Progressive determination during formation of the anteroposterior axis in *Xenopus* laevis. *Cell* 58 171–180. 10.1016/0092-8674(89)90413-3 2752418

[B35] Slack J. M. W. Beck C. W. Gargioli C. Christen B. (2004). Cellular and molecular mechanisms of regeneration in *Xenopus*. *Philos. Trans. R. Soc. Lond. B Biol. Sci.* 359 745–751. 10.1098/rstb.2004.1463 15293801 PMC1693370

[B36] Tereshina M. B. Ermakova G. V. Ivanova A. S. Zaraisky A. G. (2014). Ras-Dva1 small GTPase regulates telencephalon development in *Xenopus* laevis embryos by controlling Fgf8 and Agr signaling at the anterior border of the neural plate. *Biol. Open* 3 192–203. 10.1242/bio.20147401 24570397 PMC4001240

[B37] Tereshina M. B. Ivanova A. S. Eroshkin F. M. Korotkova D. D. Nesterenko A. M. Bayramov A. V. (2019). Agr2-interacting prod1-like protein Tfp4 from *Xenopus* laevis is necessary for early forebrain and eye development as well as for the tadpole appendage regeneration. *Genesis* 57:e23293. 10.1002/dvg.23293 30912273

[B38] Tseng A.-S. Levin M. (2008). Tail regeneration in *Xenopus* laevis as a model for understanding tissue repair. *J. Dent. Res.* 87 806–816. 10.1177/154405910808700909 18719206 PMC10445233

[B39] Tseng A.-S. Beane W. S. Lemire J. M. Masi A. Levin M. (2010). Induction of vertebrate regeneration by a transient sodium current. *J. Neurosci.* 30 13192–13200. 10.1523/JNEUROSCI.3315-10.2010 20881138 PMC2965411

[B40] Tsuji T. Satoyoshi R. Aiba N. Kubo T. Yanagihara K. Maeda D. (2015). Agr2 mediates paracrine effects on stromal fibroblasts that promote invasion by gastric signet-ring carcinoma cells. *Cancer Res.* 75 356–366. 10.1158/0008-5472.CAN-14-1693 25488752

[B41] Zhu Q. Mangukiya H. B. Mashausi D. S. Guo H. Negi H. Merugu S. B. (2017). Anterior gradient 2 is induced in cutaneous wound and promotes wound healing through its adhesion domain. *FEBS J.* 284 2856–2869. 10.1111/febs.14155 28665039

