# Supplementary material for: The Secreted Protein Disulfide Isomerase Ag1 Lost by Ancestors of Poorly Regenerating Vertebrates Is Required for Xenopus laevis Tail Regeneration
Source: Front Cell Dev Biol. 2021 Oct 5;9:738940. doi: 10.3389/fcell.2021.738940 (PMC8523854; doi:10.3389/fcell.2021.738940)
Supplement: Supplementary file 1 [file Table_1.doc]

**Supplementary material**

**Primers for qRT-PCR:**

| *fgf8* | *dir -5’ -CTGCGTCTTCTCGGAAATTGTC, rev - 5’-TTTGGCAACCTCTTCATGAAGT* |
| --- | --- |
| *msx1b* | *dir -5’ -TCTCCTATGGGACTTTACACA, rev - 5’- AATCACTCAAGTCTTCTTTC* |
| *fgf20* | *dir -5’ -ATCACAGCCGATTCGGTATCC, rev - 5’-CTCAAACTGTTCCCGAAAAATGC* |
| *ef-alfa* | *dir -5’ - AAGAACGTGTCAGTCAAGGACAT, rev - 5’ - CGTAACCCTGAGAGATCTGACCA* |
| *odc* | *dir -5’ - CTCCACCTTCAATGGCTTCCAG, rev - 5’ - AGTGGGATGGCACGTTTCCAG* |
| *cyclin d1* | *dir-5’- AGCAGCAACACAACGCTTC, rev - 5’ - GATCCGTTTGATGTCAGATG* |
| *cdk4* | *dir -5’ - CGTATTTCCGCCTCCGAT, rev - 5’ - ATGCCGTTTCATTTCACTT* |
| *cdca9* | *dir -5’ - CTGACTTTGACTTATGGAATC, rev - 5’ - ATTAGTACACAAAGCCATTA* |
| *ag1* | *dir 5′-TGCTGCCAAGTCTGAGCCTGC, rev- 5′ - TCCTGAGCCAGTTTCTGTGCCA* |
| *agr2* | *dir 5′-TGGCCAGTATGTTCCCAAGGTTGT, rev 5′ - CATCACTTTAGCATACACCTCCGC* |

Primers for the *odc, ef-1alpha, fgf20a, ag1, agr2, fgf8, msx1b* were the same as in (Ivanova et al. 2018; Ivanova et al. 2013).
PCR efficiency (PE) for *cyclin d1, cdk4, cdca9* was calculated as described previously and were respectively the following: 1,903, 1,875, 1,732.

**Primers for cDNA synthesizing:**

| *cyclin d1 forward* | *5’ - CGCAGGATCCCACAGACAACATGGAGCTT* |
| --- | --- |
| *cyclin d1 reverse* | *5’ - TGTGATCGAATTCATGTCAGATGTTGACGT* |

**Morpholino** oligonucleotides (MO)

| control vivo-MO | *5’ GCAAGATTCCTCATTCAAAAGTCTC* |
| --- | --- |
| *agr2* vivo-MO | *5’ CAGTGCTTTACTCCAGAGGCAGGAG* |
| *ag1* vivo-MO | *5’ TCTGTGGATGTCTTGCTCTTCCAGG* |
| *ag1* MO | *5’ TCTGTGGATGTCTTGCTCTTCCAGG* |
| *agr2* MO | *5’ CAGTGCTTTACTCCAGAGGCAGGAG* |
| control MO | *5’ TTAACTGTTTAATGTTGAATGAGAAC* |
| *ag1* photo-MO | *5’ GGAAGAGCAPGACATCCACAG* |
| *agr2* photo-MO | *5’ GCCTCTGPAGTAAAGCACTG* |

**Morpholino Oligonucleotides (MO) sequences and efficiency/specificity tests**

See scheme of MO sites on *ag1* and *agr2* mRNAs on **Figure S1A**.

The evaluation the effectiveness and specificity of *ag1* and *agr2* MO is published in our previous article (Tereshina et al. 2014). Here, we tested the efficiency and specificity of vivo- and photo-MO. For this, we injected *Xenopus* two-cell embryos with the following solutions: *ag1-TagRFP* mRNA or *agr2-TagRFP*  (which were obtained earlier (Tereshina et al. 2014)) (100pg/blastomere ) alone or in a mixture with agr-specific or control vivo-MO/(photo-MO+MO). Embryos injected with photo-MO were illuminated for 30 minutes by 365 nm light immediately after injection and incubated at daylight or as control were injected under 560mn and kept in dark. At the blastula-early gastrula stage, the injected embryos were collected and analyzed for the presence of Ag1-tagRFP and Agr2-tagRFP proteins by Western blotting with anti-tRFP antibody (Evrogen, cat.#AB233) as described previously. Tubulin was detected by monoclonal anti-tubulin antibody, obtained from the Institute of Protein RAS, and was used as a loading control. Strong suppression of *ag1-tagRFP* and *agr2-tagRFP* mRNAs translation was observed in embryos microinjected with these mRNAs and the respective *ag1* vivo-MO and *agr2* vivo-MO (**Figure S1B and C**). In contrast, co-injection of control vivo*-*MO did not show any Ag1-tagRFP or Agr2-tagRFP translation inhibition. Similar results were observed for cross-injection of *ag1-tagRFP* RNA with *agr2* vivo-MO or *agr2-tagRFP* RNA + *ag1* vivo-MO. These results confirm the efficiency and specificity of *ag1 vivo-MO* and *agr2 vivo-MO.* The testing of photo-MO efficiency showed that co-injection of *ag1-tagRFP* RNA with *ag1*(photo-MO+MO) does not disturb translation until illumination with 365nm light, which leads to *ag1* photo-MO disruption and *ag1* MO release and action preventing mRNA translation (**Figure S1D**).

**Recombinant proteins**

For the production of the *Xenopus laevis* recombinant Ag1 and Agr2 proteins, we used the *E.coli* expression system. The Agr2.L expressing construct was generated previously on the base of pQE80, in which the cassette encoding 6His, the site for TEV proteinase and *agr2* cDNA reading frame, was inserted (Ivanova et al. 2018). The *ag1.s* cDNA coding frame was sub-cloned into this *pQE80-6His-TEV-ag1* construct instead of *agr2*. The overnight cultures of DH5 *E. coli* strain transformed with these plasmids were prepared. To induce protein production, IPTG (isopropyl-β-D-1-thiogalactopyranoside) was added to the medium at a final concentration of 1 mM and incubated for 4 h. After incubation, the mixture was centrifuged for 10 min at 1400 g, the supernatant was discarded, and 15 ml of the lysis buffer (50 mM NaH2PO4, 300 mM NaCl, 1 μM PMSF, 1 mg/mL lysozyme, pH 8.0) was added to 0.7-1g of the pellet. The recombinant protein purification was carried out on ice according to the recommendations for the use of the TALON® Metal Affinity Resins from the User Manual (Clontech Laboratories, Inc. A Takara Bio Company). The resulting fraction was dialyzed against 2 L 1× PBS for 12 h. To remove 6His-tag, we added 10 units of recombinant 6 His TEV proteinase (GeneScript) (typical ratio 1 V TEV: 100 V solution) to the dialyzed protein and incubated overnight at 4^o^C. After incubation, the removal of proteinase and uncleaved protein was performed by incubation with 50 μL of TALON resin. As a result, from 1g of the *E. coli* pellet, we usually obtained 0.25-0.3 mg of purified Ag1 protein and 0.5-0.6 mg of Agr2 protein. The purity of the obtained proteins was checked using SDS-PAGE analysis and the efficiency of His-tag removal by immunoblotting with monoclonal anti-6HIS AP-conjugated antibodies. Analysis of the stability of the Agr2 and Ag1 proteins was carried out by SDS PAAG and SuperBlue Coomassie staining. Samples were prepared with protein precipitation from 1 ml of the incubation medium as described in (Martynova et al., 2021) (**Figure S3**).


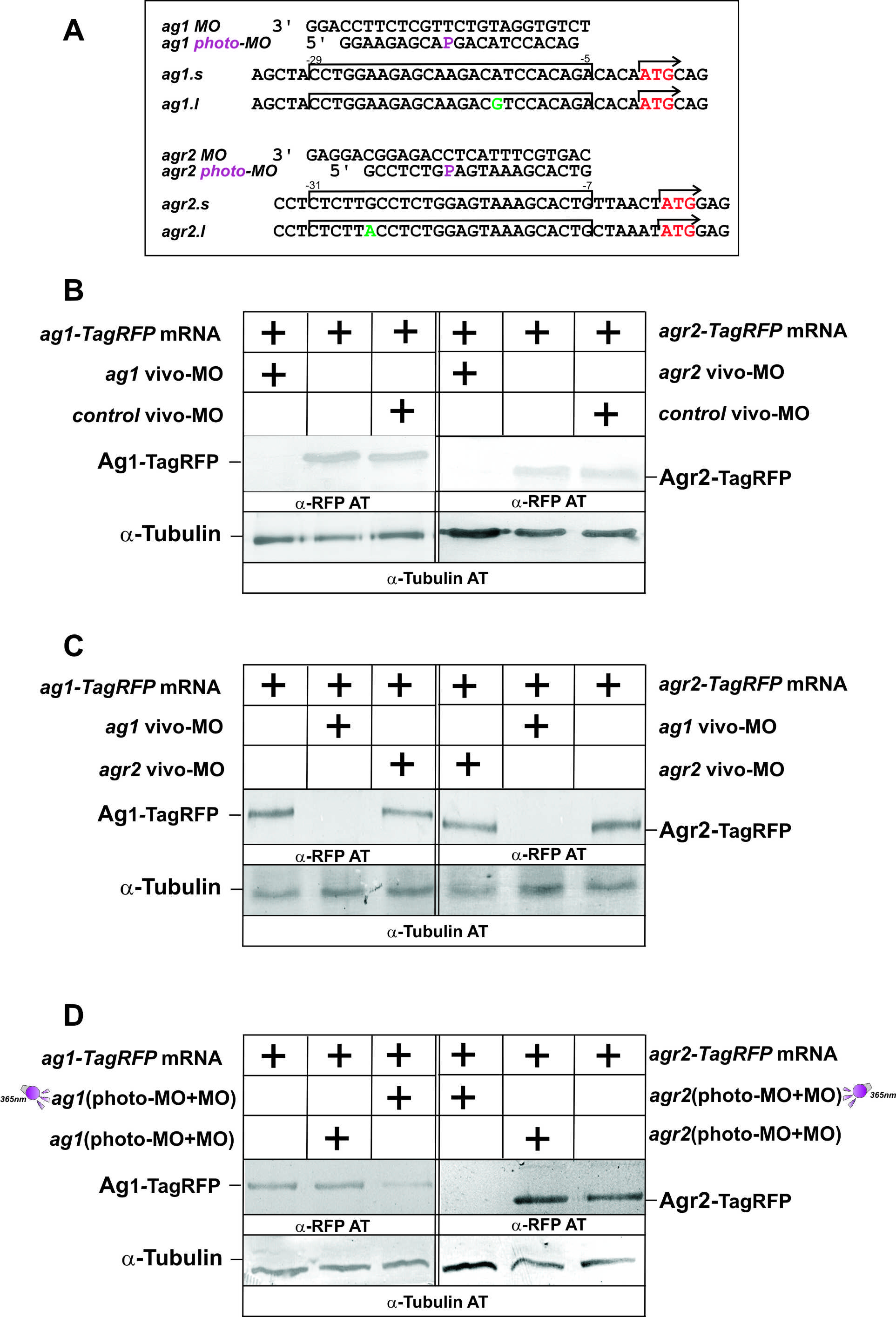


**Figure S1. Vivo- and photo-morpholino oligonucleotides (MO) efficiency tests**. **(A)** The MO (conventional and vivo-MO) and complementary photo-MO sequences and scheme of their sites on *ag1* and *agr2* mRNAs. **(B)**Results of Western blotting with rabbit anti-tRFP (Evrogen) and anti-rabbit alkaline phosphatase antibody (Sigma-Aldrich) and monoclonal anti-tubulin antibody (Sigma-Aldrich) demonstrate specific and effective inhibition of Ag1-tagRFP/Agr2-tagRFP synthesis by *ag1/agr2* vivo-MO, but not by control vivo-MO. **(C)** Results of Western blotting demonstrate specific and effective inhibition of Ag1-tagRFP synthesis by *ag1 vivo-MO*, but not by *agr2 vivo-MO* and vice versa. **(D)** Results of Western blotting demonstrate specific and effective inhibition of Ag1-tagRFP or Agr2-tagRFP synthesis by *ag1* (photo-MO+MO)or *agr2* (photoMO+MO) only after illumination at 365 nm (violet lamp).


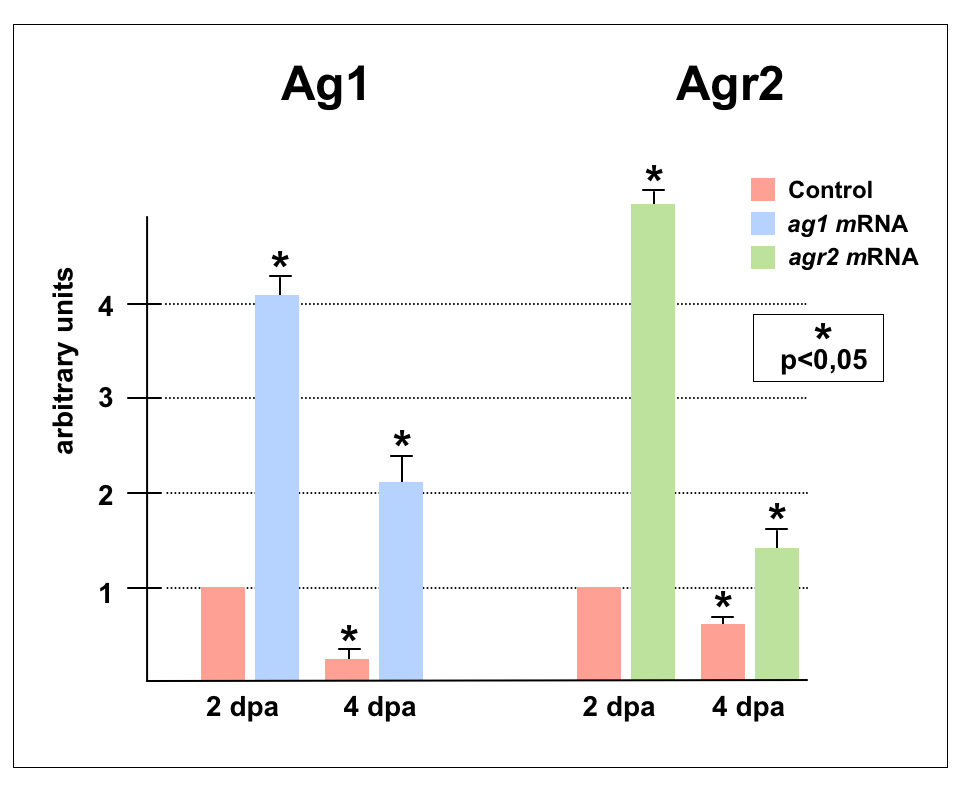

**Figure S2. Tail tips of tadpoles developed from embryos injected with *ag1* and *agr2* mRNAs contain high level of these mRNAs in the refractory period.**

The qRT-PCR analysis of *ag1* and *agr2* expression in the tips of tails amputated in the refractory period demonstrates much higher level of *ag1* and *agr2* mRNAs in the tips of tadpoles developed from embryos injected with these synthetic mRNA comparing to the tips of control tadpoles. Statistical significance was determined by t-test for independent samples, p<0.05 (asterisk). Error bars indicate SD.


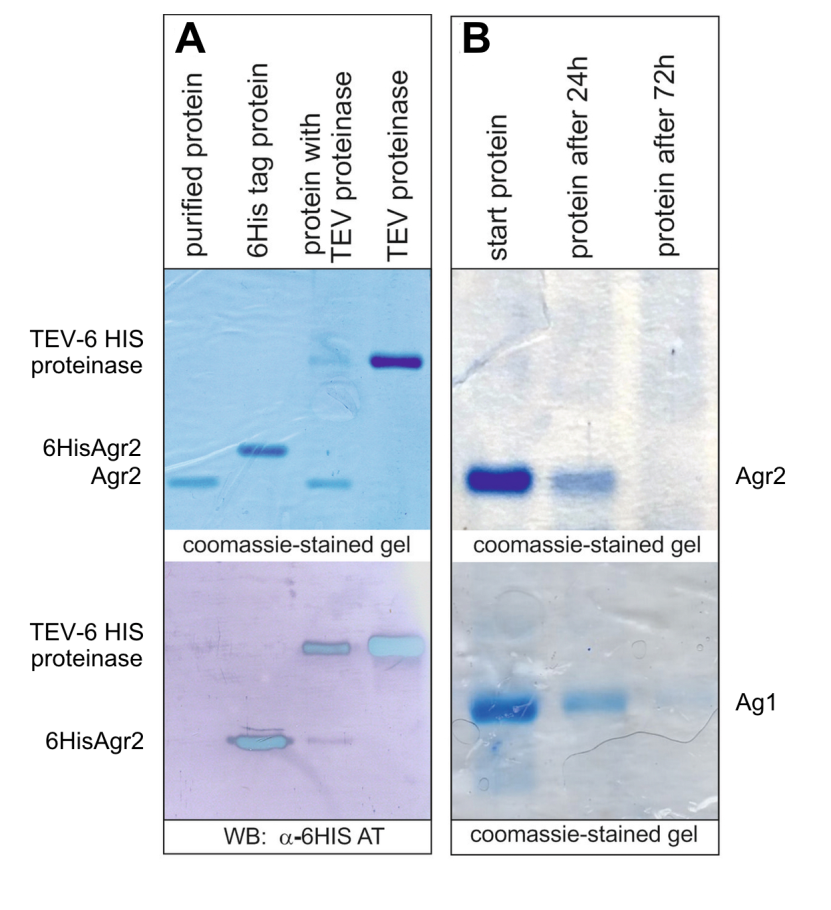


**Figure S3. Analysis of recombinant proteins. (A)** The main stages of protein purification are shown in the example Agr2. **(B)** Recombinant Ag1 precipitated from 1ml of 0.1XMMR after incubation with tadpoles for 0, 24 and 72 hours, respectively (Martynova et al., 2021).

**
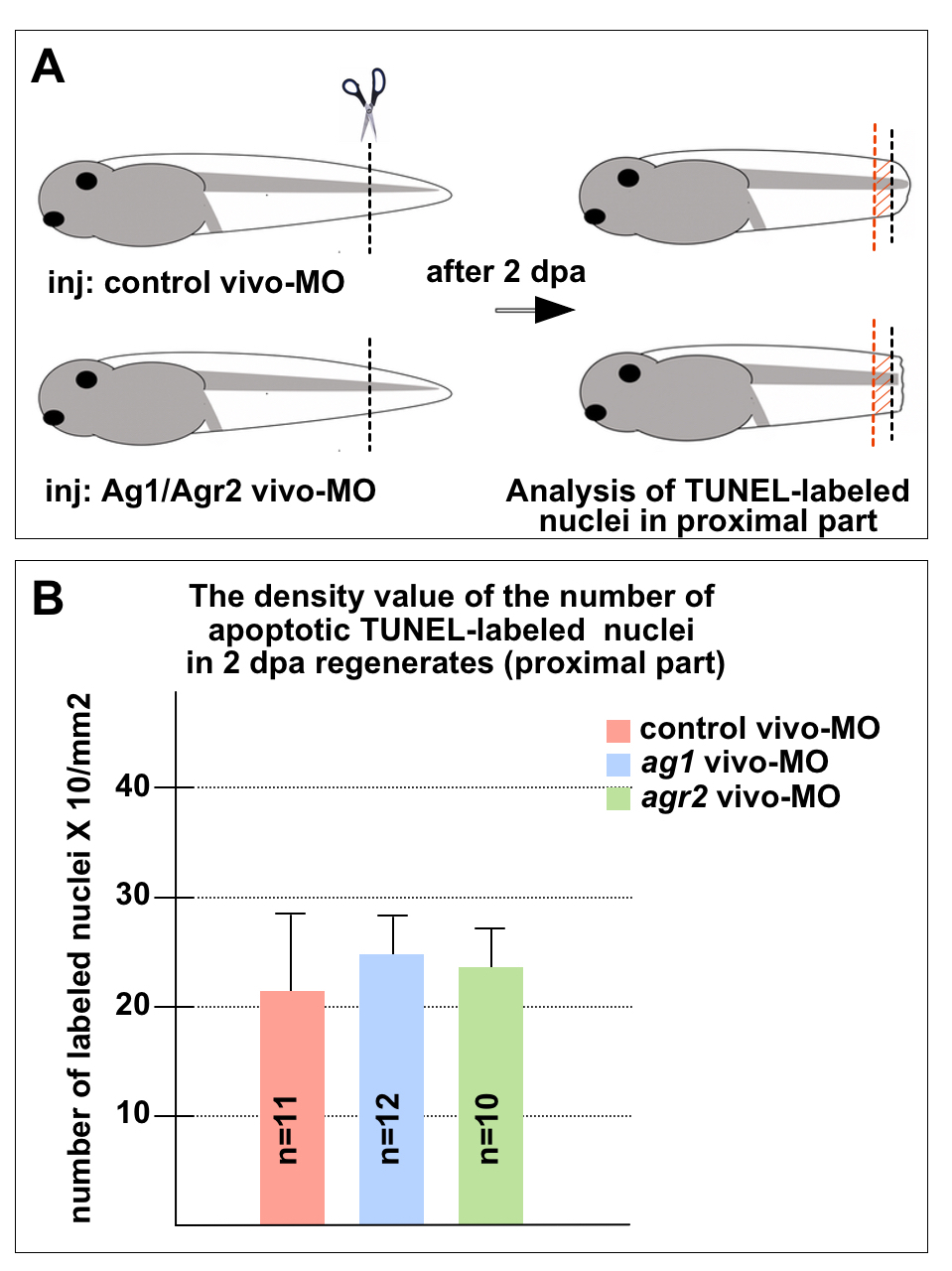
**

**Figure S4. The apoptotic cell density in the area proximal to amputation level does not change in response to *ag1* and *agr2* downregulation.** (A) scheme of the experiment: tadpoles tail stumps at st.40-41 were injected by control vivo-MO, *ag1-*vivo MO  or *agr2-*vivo-MO. After 2 days, the tadpoles' tails were stained for the presence of apoptotic cells using the TUNNEL analysis, and then in the area located proximal to the level of amputation (between the black and red lines, the distance between which was approximately equal to half the tail width), the density of apoptotic cells was determined per 1 mm^2^. (B) No statistically significant difference in the apoptotic cell density was revealed between control tails and those injected with *ag1-*vivo MO  or *agr2-*vivo-MO. Error bars indicate SD.


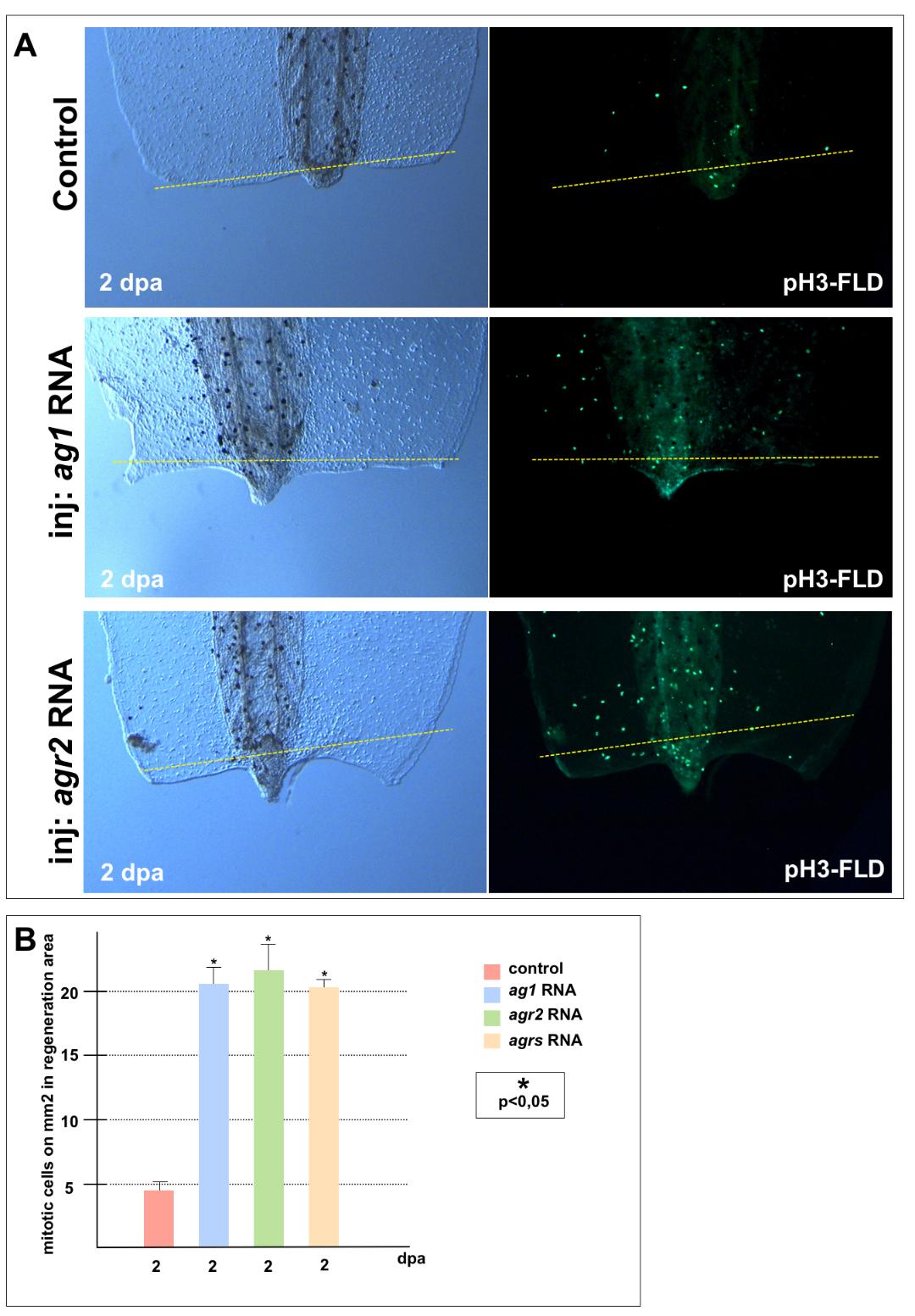


**Figure S5**. **Overexpression of *ag1/agr2* is sufficient to reactivate cell prolifertion and regeneration in the refractory period when they are naturally blocked.** **(A)** The transmitted light and fluorescent images of tail tips of tadpoles injected with solution of *RDA* amputated in the refractory period after immunostaining with anti-pH3-fluorescein demonstrate very low mitotic activity in the tail tip area at 2dpa. In contrast, tadpoles injected with the solution of *ag1 RNA* or *agr2 RNA* after amputation in the refractory period demonstrate much higher proliferative activity in distal area of the tail. **(B)** Quantification of number of mitotic cells per 1 mm^2^ of tail regenerating area. Data of five independent experiments (10 tadpoles of each injection type were used in 1 experiment) were used for statistical analysis; statistical significance was determined using t-test for independent samples, p<0.05 (asterisk). Error bars indicate SD.

**References**

Ivanova, A. S., Martynova N. Y., Komarov P. A., Orlov E. E., Ermakova G. V., Zaraisky A. G., and Tereshina M. B. (2018). Obtaining of Agr2 Specific Antibodies and Determination of the Agr2 Protein Distribution Pattern during Early Embryonic Development and Tadpole Regeneration in Xenopus Laevis. *Russian Journal of Developmental Biology* 49 (6): 393–97. doi: 10.1134/s1062360418060036.

Ivanova A. S., Korotkova D. D., Ermakova G.V., Martynova N. Y., Zaraisky A. G., and Tereshina M. B. (2018). Ras-Dva Small GTPases Lost during Evolution of Amniotes Regulate Regeneration in Anamniotes. *Scientific Reports* 8(1): 13035. doi: 10.1038/s41598-018-30811-0.

Ivanova A. S., Tereshina M. B., Ermakova G. V., Belousov V. V., and Zaraisky A. G. (2013). Agr Genes, Missing in Amniotes, Are Involved in the Body Appendages Regeneration in Frog Tadpoles. *Scientific Reports* 3: 1279. doi: 10.1038/srep01279.

Martynova N. Y., Parshina E. A., and Zaraisky A. G. (2021). Protocol for Separation of the Nuclear and the Cytoplasmic Fractions of Xenopus Laevis Embryonic Cells for Studying Protein Shuttling. *STAR Protocols* 2 (2): 100449. doi: 10.1016/j.xpro.2021.100449.

Tereshina M. B., Ermakova G. V., Ivanova A. S., and Zaraisky A. G. (2014). Ras-Dva1 Small GTPase Regulates Telencephalon Development in Xenopus Laevis Embryos by Controlling Fgf8 and Agr Signaling at the Anterior Border of the Neural Plate. *Biology Open*, 3(3): 192-203. doi: 10.1242/bio.20147401.
